# Concurrent Prolapsed Omphalocele and Giant Hepatic Cyst Initially Suspected as an Umbilical Cord Cyst in the Prenatal Period: A Case Report

**DOI:** 10.70352/scrj.cr.24-0006

**Published:** 2025-09-19

**Authors:** Yuta Takeuchi, Seiichiro Inoue, Yuki Muta, Keisuke Sawada, Taisuke Hayashi, Kohei Kawaguchi, Akio Odaka

**Affiliations:** 1 Department of Pediatric Surgery, Saitama Medical Center, Saitama Medical University, Kawagoe, Saitama, Japan; 2 Department of Pathology, Saitama Medical Center, Saitama Medical University, Kawagoe, Saitama, Japan

**Keywords:** omphalocele, hepatic cyst, umbilical cord cyst, Beckwith–Wiedemann syndrome

## Abstract

**INTRODUCTION:**

Umbilical cord cysts detected after the 2nd trimester of pregnancy are associated with a variety of complications, including omphalocele, and thus require careful monitoring. Congenital hepatic cysts are rare, as are reports of their coexistence with omphaloceles. Herein, we present an unusual case of an omphalocele complicated by a large hepatic cyst that was initially suspected to be an umbilical cord cyst during the fetal period.

**CASE PRESENTATION:**

A male infant was delivered via caesarean section at 36 weeks of age, with ruptured membranes and cloudy amniotic fluid. Fetal ultrasonography at 16 weeks had previously revealed an omphalocele with intestinal prolapse, and at 20 weeks, an umbilical cord cyst was suspected. At birth, the herniation sac ruptured, and abdominal wall closure and cyst excision were performed. The cyst, initially thought to be umbilical in origin, was instead identified as a hepatic cyst connected to the liver. Pathological examination confirmed the diagnosis. The infant was further diagnosed with Beckwith–Wiedemann syndrome, and was ultimately discharged 32 days postoperatively, with no recurrence to date.

**CONCLUSIONS:**

Although asymptomatic congenital liver cysts can often be managed conservatively with follow-up, we considered surgical intervention necessary in this case due to the presence of an omphalocele associated with suspected Beckwith–Wiedemann syndrome

## INTRODUCTION

Umbilical cord cysts are rare abnormalities commonly detected on fetal ultrasound examinations. Approximately 3% of cysts are identified in the 1st trimester of pregnancy, about half of which resolve spontaneously.^1)^ However, when umbilical cord cysts persist beyond 15 weeks of pregnancy, the possibility of an abnormality must be considered.^1–3)^

Congenital hepatic cysts are rare and generally asymptomatic unless complications such as rupture, hemorrhage, respiratory symptoms due to progressive cyst enlargement, or torsion occur.^4)^ Surgical resection may be indicated in some cases, even in pediatric patients, particularly when respiratory symptoms induced by diaphragmatic elevation, hepatomegaly, or malignancy are suspected.^4)^ However, reports on neonatal hepatic cysts complicating omphalocele are rare.

In this report, we present a case of an omphalocele complicated by a large hepatic cyst that was initially suspected to be an umbilical cord cyst during the fetal period.

## CASE PRESENTATION

**Fig. 1:**
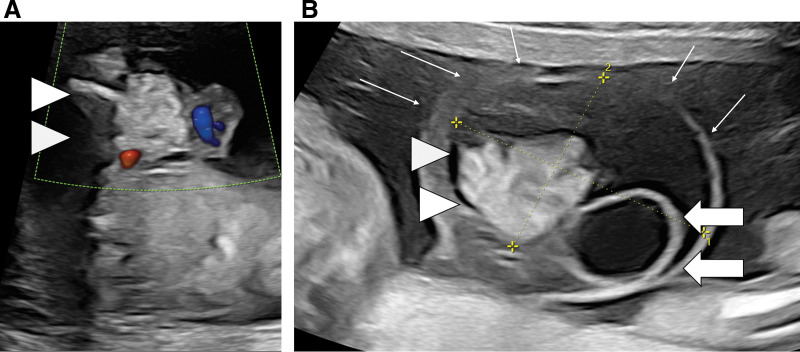
Fetal ultrasound findings. (**A**) Fetal ultrasound performed at 16 weeks 3 days gestation, showing an intestinal prolapse within the umbilical cord (arrowhead). (**B**) Fetal ultrasound at 20 weeks 3 days gestation, showing an omphalocele measuring 48 × 34 mm (thin arrow), an intestinal prolapse (arrowhead), and a large umbilical cyst measuring 18 × 16 mm (arrow).

**Fig. 2:**
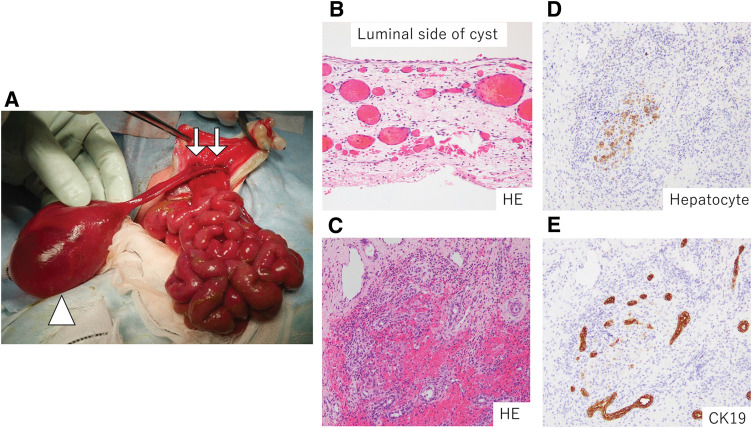
Surgical findings and results of surgical specimen pathology immediately after birth. (**A**) The prolapsed organs included most of the small intestine, the colon, and the cystic lesions. No Meckel’s diverticulum was observed. The cystic lesion, measuring 50 × 50 × 40 mm (arrowhead), was intraperitoneal in a cord-like fashion and communicated with the left side of the liver (arrow). (**B**) Histopathological image of the cyst wall (cross section). The upper side shows the luminal surface, while the inner surface of the cyst is lined by a single layer of flattened epithelial cells. The cyst wall was fibrous, with scattered vascular formations, but no ovarian-like stroma. (**C**–**E**) Pathological findings in the section of the cordate. Hepatocytes showing Hepatocyte (+) and bile ducts showing CK19-positive cells were sporadically distributed in the cordate, supporting the hypothesis that the tissue was of hepatic origin. CK19, CK1 cytokeratin; HE, hematoxylin and eosin

The patient was a male infant delivered via caesarean section at 36 weeks and 1 day of gestation, owing to complete water breakage and amniotic fluid clouding, with a birth weight of 3598 g. His 1- and 5-min Apgar scores were 6 and 8, respectively. Abdominal wall abnormalities were suspected on fetal ultrasonography at 16 weeks and 2 days of gestation. At 16 weeks and 3 days of gestation, the patient was diagnosed with an omphalocele (**Fig. 1A**). At 20 weeks and 3 days of gestation, an umbilical cord cyst, suggestive of patent urachus, was suspected (**Fig. 1B**). Other differential diagnoses included umbilical cord varicocele, umbilical cord hemangioma, urachal cyst, and yolk intestinal cyst. The cyst progressively increased in size during the course of the pregnancy. At birth, the umbilical herniation sac had ruptured during the emergency caesarean section, and intestinal prolapse was observed. Abdominal wall closure was performed on day 0. The abdominal wall defect was 3 cm in size, and the prolapsed organs primarily comprised the small intestine and colon. The cystic mass, which was initially suspected to be an umbilical cord cyst during the fetal period, comprised a cord-like structure, in which the pedicle was contiguous with the left lobe of the liver (**Fig. 2A**). No cystic communication was observed. A clinical diagnosis of hepatic cyst was made, and ligation and dissection were subsequently performed on the cystic side. The herniated organs were returned, and 1-stage abdominal wall closure was performed. Excess amniotic fluid remained, while Steri-Strip reinforcement was applied to protect the closed area. Pathological examination revealed that the inner surface of the cyst was lined with a single layer of flattened epithelial cells, while the cord-like pedicular portion revealed a tissue of hepatic origin, which was diagnosed as a hepatic cyst (**Fig. 2B**–**2E**). Perioperatively, a giant tongue, neonatal hypoglycemia, and gigantism were identified, and genetic testing performed at a later date also diagnosed the patient with Beckwith–Wiedemann syndrome. The patient was discharged on POD 32.

## DISCUSSION

Umbilical cord cysts have been identified using ultrasonography at various stages of gestation. Ghezzi et al. previously reported that all children with a single umbilical cord cyst detected during early pregnancy were born without complications.^5)^ The incidence of umbilical cord cysts after 14 weeks of gestation has not been well established; however, these cysts are believed to be associated with complications such as omphalocele and chromosomal abnormalities. Other differential diagnoses for umbilical cysts included umbilical varicocele, umbilical hemangioma, urachal cyst, and yolk intestinal cyst.^1,2,6)^ In the present case, a single umbilical cord cyst was suspected during the 2nd trimester at 16 weeks’ gestation. As previous reports have suggested, in cases in which an umbilical cord cyst is identified after the 2nd trimester, it is essential to consider the possibility of associated complications.

The incidence of congenital hepatic cysts is approximately 2.5% of live births, with a much lower incidence in prenatal cases, most of which are detected incidentally on prenatal imaging studies.^4,7,8)^ These cysts are more common in girls and are generally simple in nature, with approximately 80% originating from the right lobe of the liver.^7)^ Congenital hepatic cysts can be intrahepatic, partially extrahepatic, or circumscribed, and are generally asymptomatic.^7,9)^ However, there have been reports of respiratory symptoms developing in cases of large cysts, and caution is generally advised when the cysts exceed 4 cm in diameter.^4,10,11)^ Consequently, surgical intervention is indicated when cysts are sufficiently large to cause symptoms.^4)^ Histopathologically, hepatic cysts are lined with a monolayer of columnar, cuboidal, or squamous epithelium cells.^12,13)^ In the present case, the cyst lumen was lined by a monolayer of squamous epithelial cells, but was determined to be of hepatic tissue origin due to positivity for hepatocyte and cytokeratin 19 markers. As such, the patient was pathologically diagnosed with a hepatic cyst.

Beckwith–Wiedemann syndrome is a genetic disease complicated by tumors. Previous studies on patients with Beckwith–Wiedemann syndrome have reported hepatic cysts.^14,15)^ However, omphalocele complicated by hepatic cysts has been reported in only 2 previous cases of Beckwith–Wiedemann syndrome, and only 1 other case has been reported in association with multiple malformations.^15–17)^ Thus, hepatic cysts complicated by omphaloceles, as in the present case, have rarely been reported. Despite the ongoing debate regarding surgical management, surgery was chosen in this case because of the known complications of Beckwith–Wiedemann syndrome,^14,15,18)^ including the potential for tumor development, the large cyst size, and the possibility of future symptoms, as well as the risk of torsion due to the herniated nature of the cyst. Although the decision to perform surgery can be challenging and case-dependent, resection of hepatic cysts may be considered necessary, even in rare cases such as this.

## CONCLUSIONS

Congenital hepatic cysts are rare and typically asymptomatic; however, large cysts may require surgical intervention. In this case, the hepatic cyst, complicated by an omphalocele, required surgical treatment because of the patient’s associated risk factors, including Beckwith–Wiedemann syndrome. The management of omphalocele in patients with suspected Beckwith–Wiedemann syndrome, particularly when complicated by hepatic cysts, necessitates a comprehensive strategy that includes surgical intervention.
